# News and Coming Events

**Published:** 1908-03-28

**Authors:** 


### NEWS AND COMING EVENTS.

Dr. Mierzejewski, physician to the Emperor of Russia, died in Paris on March 18.

Mr. Cecil Rowntree, M.B., B.S., F.R.C.S., has been appointed registrar to the cancer wards of the Middlesex Hospital.

A claim of £4 a week for five weeks has recently been paid under the provisions of the "Doctor's Own Policy," in connection with a bicycle accident to a medical practitioner resident in Yorkshire.

Dr. George Henry Falkiner Nuttall, F.R.S., Quick Professor of Biology in the University of Cambridge, has been elected to a professorial Fellowship at Magdalene College.

Mr. John Poland, F.R.C.S., late senior surgeon to the City Orthopædic Hospital, has resigned the post of surgeon to the Royal National Orthopædic Hospital, to which he was recently elected.

The committee for the removal of King's College Hospital to South London has received a cheque for £1,000 from the Goldsmiths' Company, this being its second special donation in aid of the fund.

Dr. James Kerr, medical officer (education), L.C.C., will deliver two lectures on "Physical Conditions Affecting Health in Schools," at the Royal Sanitary Institute, Parke's Museum, Margaret Street, Regent Street, W., on Mondays, April 6 and 13, at 7 p.m.

Owing to the deaths of Drs. Robert and Fancourt Barnes, consulting physicians to the Royal Maternity Charity of London, Dr. Henry Gervis and Dr. George Ernest Herman have been unanimously elected by the committee to fill the vacant posts.

Dr. John C. Thresh, M.D., D.Sc., D.P.H., will deliver a lecture on "The Milk Problem" in the Medical Theatre, Birmingham University, on Monday, March 30, at 8 p.m. The lecture will be under the auspices of the Midland Counties Branch of the Incorporated Institute of Hygiene.

The Earl Cawdor, the Treasurer of the London Homœopathic Hospital, has received on behalf of the hospital £1,000, a legacy from the estate of the late Mrs. Elizabeth Mason, of St. Leonards-on-Sea; also £1,300, a legacy from the estate of the late Miss Emily Rebecca Leon, to endow a bed in a female ward, to be called "The Leon Bed."

A laboratory in connection with the Liverpool Hospital for Consumption and Diseases of the Chest in Mount Pleasant was opened on March 24 by the Lord Mayor (Dr. R. Caton. The total cost of the laboratory and equipment will amount to about £1,260.

The annual meeting of the Chelsea Hospital for Women was held on March 19, the President, Lord Glenesk, in the chair. The Chairman stated that the most satisfactory feature of the report was the low death-rate, of less than 1½ per cent., with which they had been able to get through a year's medical work of exceptional difficulty. This was due to the great skill and care of the medical and nursing staffs, and the almost supercare with which all aseptic precautions were carried. The amount of sterilising necessary threw a great responsibility on the nursing staff and tended, among other things, materially to increase the cost per patient.

Dr. V. Zachary Cope has been appointed Surgical Registrar to the London Temperance Hospital.

The Council of the British Association for the Advancement of Science has nominated Prof. J. J. Thomson, F.R.S., Cavendish Professor of Experimental Physics in the University of Cambridge, to be President of the meeting of the Association which is to be held at Winnipeg next year.

Dr. Robert Henry Scanes-Spicer, who has completed 20 years' service at St. Mary's Hospital as surgeon for diseases of the throat, has received from the Board of Governors a resolution of thanks for his valuable services to the institution, and an intimation of his election as consulting surgeon for these diseases.

A provincial sessional meeting of the Royal Sanitary Institute will be held at the Town Hall, Hull, on Saturday, April 4, at 11 a.m., when a discussion will take place on "Diphtheria in Elementary Schools and its Prevention," which will be opened by Dr. J. Wright Mason. The chair will be taken by Colonel J. Lane Notter, M.A., M.D., R.A.M.C., Deputy-Chairman of the Council of the Institute.

According to a correspondent of the *Times*, the late John Joseph Ayre, M.R.C.S., of Colne, Lancashire, who died recently in his ninety-seventh year, was the oldest medical man in England. He was born on May 19, 1811, and had thus lived during five reigns. He was educated at King's College, and qualified in 1832. Mr. Ayre took an active part in local affairs, and retained to the end his interest in scientific and medical progress.

The arrangements for the forthcoming concert of the League of Mercy are progressing favourably. The King and Queen, the Prince and Princess of Wales, and other members of the Royal Family will be present at the entertainment, which is to take place at the Royal Albert Hall on Saturday, May 30, at 3.30 p.m. Among the artistes who have offered to sing are Mme. Melba and Signor Caruso, while the Royal Amateur Orchestral Society have also offered their services.

In the unavoidable absence of the Marquis of Zetland, President of the institution, Mr. C. D. Rudd took the chair at the annual Court of Governors of the Mount Vernon Hospital for Consumption and Diseases of the Chest. The report, which was adopted on the motion of the Chairman, showed a satisfactory year's working. The number of in-patients at the Hampstead Hospital had increased by 26, and the number of beds in daily occupation by five; while at Northwood the number of in-patients had increased by 107, and the number of beds in daily occupation by 22.

The Right Hon. the Earl of Derby, K.G., President, presided at the Annual Court of Governors of the Brompton Hospital for Consumption held on March 13. The report of the Committee of Management for the past year stated that 273 patients had been transferred from the wards at Brompton to the Sanatorium at Frimley, where, as the result of treatment by carefully graduated exercise, the majority have experienced complete arrest of the disease and have been enabled to return to their former occupations. The report showed that 204 *x*-ray examinations had been made at the Hospital, which had again proved most valuable as an aid to diagnosis, and that much useful work had been carried out in the laboratories in the examination of the Opsonic Indices of the patients.

The Queen has given permission for the North-Eastern Hospital for Children, Hackney Road, to be in future known as "The Queen's Hospital for Children." A special meeting of the Governors of the institution will be held on April 2, at 4 P.M., to receive this information formally and give effect to the change of name.

Lord Alverstone, the Lord Chief Justice, presided at the annual meeting of the London Temperance Hospital on March 23. Lord Alverstone said they could look back to the past year with great satisfaction; the splendid hall for out-patients had been opened by the Duchess of Argyll, who had since become patron of the institution; the buildings had been improved and everything had been brought up to date. With increased accommodation a larger staff became necessary, and funds for maintenance would continue to be required. He was much struck with the statement in the report that during thirty-four years the in-patients had numbered 27,226, and it had only been considered needful to make trial of alcohol in eighty cases. It was thus clear that whatever theoretical value some might attach to alcohol as a drug, it could be almost entirely and safely dispensed with, while the patients themselves enjoyed immunity from those moral hazards to which many of them would be exposed by imbibing alcohol in quantities, however small.

